# Modeling and simulation of extended ant colony labor division for benefit distribution of the all-for-one tourism supply chain with front and back decoupling

**DOI:** 10.3389/fbioe.2023.985550

**Published:** 2023-03-30

**Authors:** Rundong Liao, Shufang Li, Conghui Wu, Xiang Zhang, Changbing Jiang, Ruolan Li

**Affiliations:** ^1^ School of digital Commerce and Trade, Zhejiang Institute of Mechanical and Electrical Engineering, Hangzhou, China; ^2^ School of Accounting and Finance, Zhejiang Vocational College of Commerce, Hangzhou, China; ^3^ School of Management Engineering and E-commerce, Zhejiang Gongshang University, Hangzhou, China; ^4^ School of Economics and Trade, Chengdu Agricultural College, Chendu, China

**Keywords:** all-for-one tourism, division of labor in ants, front-background decoupling, extended ant colony labor division model, benefit distribution

## Abstract

This paper takes the supply chain alliance under the decoupling of the front and back of the all-for-one tourism as the research object. Considering the three behavior stimuli of self-benefit, altruism, and invariance, this article resets the attributes such as environmental stimuli and response threshold of ants based on the characteristics of the all-for-one tourism supply chain with shared services as the core under the decoupling of the front and back. Moreover, it introduces dual intervention factors to coordinate the benefit distribution process of different member companies, takes fairness as the main goal of benefit distribution, introduces relative deprivation as the measure index of fairness, and establishes a dynamic all-for-one tourism supply chain alliance benefit distribution model. The experimental results show that the extended model has good flexibility of benefit distribution and realizes the fair distribution of supply chain benefits.

## 1 Introduction

In recent years, rural tourism has become an important model of China’s tourism industry under the support of the rural revitalization strategy (Zeng and Zhuo, 2018). Nowadays, with the wide application of new technologies such as cloud computing, big data, and the Internet of Things, the smart service system has gradually improved and the smart tourism industry has gradually emerged. The rural tourism model supported by the countryside has risen to an all-for-one tourism in which cities, scenic spots, and communities are fully integrated. all-for-one tourism is a new mode of tourism which conforms to the requirement of space panorama. It aims to realize the linkage of the tourism industry with all elements, all directions, and the whole industry through the optimization and integration of comprehensive tourism resources, public service resources, and cultural resources in a certain region. As a new type of tourism, all-for-one tourism has broken through the narrow regional boundaries of the traditional scenic tourism model and has become a powerful promoter of the overall development of tourism and economic society. However, while providing the sharing service mechanism, all-for-one tourism also faces the problem that the cooperation and benefit distribution mechanisms have not been formed yet. A large number of survey results show that more than half of the strategic alliances of the tourism supply chain were forced to disintegrate because they failed to achieve the expected goals. It was found that in addition to internal and external reasons, such as strategic mistakes, insufficient technical support, and adverse market conditions, the relationship between alliances members is often mentioned by researchers. As the core of all economic activities, the establishment of an effective profit distribution mechanism has practical significance that cannot be underestimated.

In recent years, among the methods of studying the allocation problem, the swarm intelligence method with biological dynamic flexibility has attracted scholars’ extensive attention. Swarm intelligence refers to the macroscopic intelligent behaviors of social organisms such as ants, bees, and birds that have social characteristics when performing certain specific activities. It is an emergence phenomenon of simple subjects through microscopic interactions (Xiao, 2006; Xiao and Tao, 2007). Different individuals in the same social organization perform different tasks. This phenomenon is called division of labor (Waibel et al., 2006). As one of the basic models of the swarm intelligent division of labor, the ant colony labor division has been widely used in daily production and life because of its strong adaptive ability. Xiao et al. (2012a); Xiao et al. (2012b); Xiao and Wang (2016) applied the ant colony labor division model to the organization model of virtual enterprises, the multi-project scheduling of enterprise production management, and the distribution of group benefits, showing that the ant colony labor division had good adaptive distribution flexibility. Aiming at the deficiencies of the basic labor division model, Ju and Chen (2014) proposed an extended ant colony labor division model based on ability evaluation and profit-driven and applied it to the allocation of dynamic tasks. Lope et al. (2013); Castello et al. (2014); Yasuda et al. (2014) applied the ant colony labor division model to the swarm robot system, and the results showed that the ant colony labor division could achieve good self-organization. Kim et al. (2014) proposed a distributed method of the probabilistic decision-making mechanism based on the response threshold model and applied it to the search planning and task assignment of unmanned aerial vehicle (UAV) teams. In the fields of logistics, transportation, and engineering, the distribution problem based on the ant colony labor division model is to study the placement of multiple objects of different shapes and sizes in the container with the highest utilization rate (Lodi et al., 2002). Based on the ant colony labor division model, Wang et al. (2018) studied the space allocation method in polygon packaging problems, which showed good robustness of the ant division of labor. Aiming at the problem of unequal circle packing with non-deterministic polynomial difficulty, Wang and Zhang (2019) proposed a new idea of space allocation based on the group intelligence division of labor. The simulation results showed that under the stimulation–response principle of the group intelligence division of labor, the round object could choose the appropriate action to complete the adaptive space allocation task. Aiming at the traffic signal timing problem, Hu et al. (2019a); Hu et al. (2019b) designed the bee colony labor division signal timing algorithm and the bee colony dual suppression labor division algorithm. The research results showed that the excitation–suppression algorithm is effective and the double-inhibition algorithm is dynamic. All the aforementioned results showed that the distribution method based on the ant colony labor division model could better match various generalized distribution problems. However, most current literature explores the best strategies for the coordination mechanism in the tourism supply chain from the perspective of constructing different coordination models or game theory, but most models lack dynamics and are difficult to adapt to the rapidly changing market environment. Therefore, this paper considers introducing the method of group intelligence division of labor with flexible characteristics to conduct further quantitative research on the coordination of the all-for-one tourism supply chain.

## 2 Decoupling strategy of front and back of the all-for-one tourism supply chain

**FIGURE 1:**
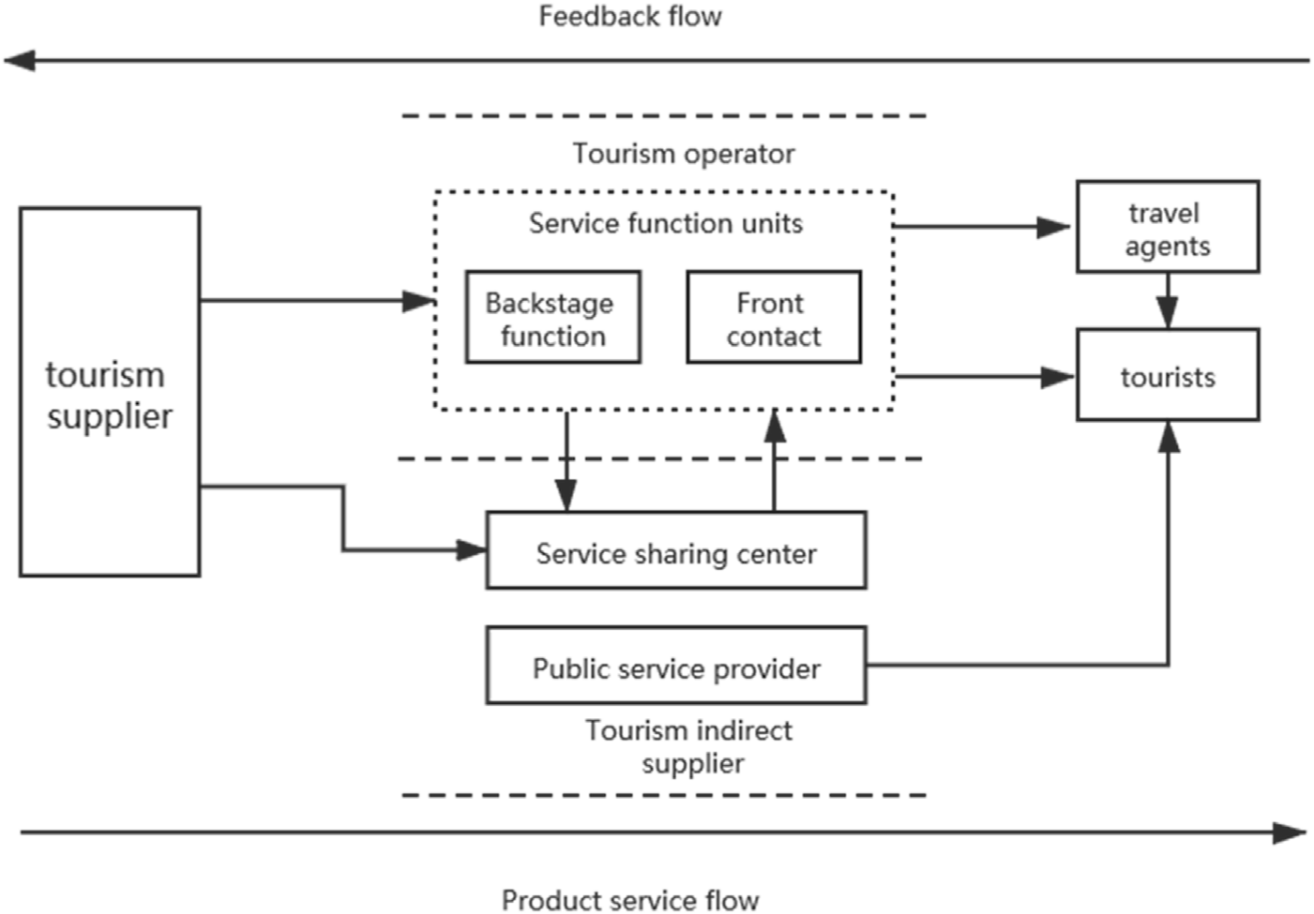
Front–background separation structure of the all-for-one tourism supply chain.

With the rapid development of information technology, transportation and storage, customer contact, and other related technologies, the mobility of backstage functions in all-for-one tourism attractions is getting stronger and stronger. Therefore, from the perspective of the overall process of all-for-one tourism, this paper introduces the separation strategy of the front and back stages in the design of the service process, reshapes its spatial structure based on the decoupling of the back stage, and divides the all-for-one tourism supply chain structure into tourism suppliers, indirect suppliers represented by service sharing centers (SSCs) (Schulz and Brenner, 2010) and public service providers, service function units, travel agents, and tourists. The specific structure is shown in Figure 1.

The structure is based on decoupling the foreground business and background functions of the service function unit to release the maximum resources and efficiency. The front and back structure is a service system spatial structure design concept adopted by service organizations to deal with customer contact in the process of service provision; that is, the service system is spatially separated into two parts: the front stage and the back stage. The front stage refers to the part of the system that directly faces customers and provides services and can be perceived by customers, while the back stage refers to the part of the system that does not directly contact customers and provides efficient and centralized support work without interference for the front stage business. Therefore, the all-for-one tourism supply chain can be extended to the procedure of suppliers–SSC–service function units–travel agencies–tourists.

The front stage of all-for-one tourism mainly includes catering and accommodation facilities, landscape sightseeing facilities, entertainment and shopping facilities, and other service function units directly facing tourists, mainly providing accommodation and catering, tourist guides, entertainment and souvenir sales, and other types of services that can be perceived by tourists. The back stage of all-for-one tourism mainly includes kitchen facilities, laundry facilities, warehouse facilities, fire-fighting facilities, drainage and sewage treatment systems, and other supportive units that are not directly oriented to tourists. It mainly provides catering semi-finished products and agricultural and sideline products’ processing, linen washing, cargo transportation and storage, fire prevention and disaster warning, emission environmental protection treatment, and other services that cannot or are difficult to be perceived by tourists.

In recent years, research on front and back structures has mainly focused on the mobility of background functions in service systems, which led to a leap from the theory of front–background structure to the theory of front–background decoupling. That is, under the premise of dividing the front stage and back stage structures, the background business is relocated or outsourced, thereby realizing the intensive and industrialized operation of background functions. At present, the front and back structure decoupling theory has been successfully applied to many service industries such as telecommunications, finance, and IT.

## 3 Problem description

In the development process of the tourism industry, there are many problems in the operation mode of the traditional supply chain. For a long time, each member enterprise in the tourism supply chain is profitable. When carrying out business activities, it only follows the principle of maximizing its own benefits and rarely considers the overall economic, social, and environmental benefits, and some member enterprises have a serious tendency of opportunism during the peak tourism seasons, which brings great efficiency loss to the whole tourism supply chain. Therefore, how to coordinate the overall benefits of the supply chain and the individual benefits among the member enterprises has become one of the core issues of tourism supply chain management.

Nowadays, with the continuous increase in the uncertainty of the tourism market environment and the change in the perception of member companies, more and more tourism suppliers and tourism operators form strategic alliances to carry out business activities together so as to achieve the goals of complementary advantages, cost reduction, information sharing, and collaborative innovation (Liu and Zhao, 2019). However, the rationality and fairness of benefit distribution in the supply chain alliance will directly affect the stability of the alliance members and are also important to maintain their continuous cooperation. Therefore, how to distribute the benefits of the supply chain alliance more reasonably needs to be solved urgently. The existing literature on the tourism supply chain benefit distribution problem is still insufficient, and the benefit distribution model is mostly a static model and is short of dynamic. Therefore, this paper considers the establishment of a dynamic benefit distribution model for the all-for-one tourism supply chain under the decoupling of front and back stages from the perspective of the alliance of tourism suppliers and tourism operators.

The benefit distribution of the all-for-one tourism supply chain alliance mainly involves three aspects: the attributes of the stakeholders, the perception of fairness by the stakeholders, and different behavior choices under the influence of internal and external conditions. The detailed description is as follows:

(1) The attributes of the stakeholders are determined by their contribution to the total benefit and their profitability. In the same tourism supply chain strategic alliance, the higher the contribution and profitability of the stakeholders, the greater their willingness to increase the benefits, and *vice versa*.
(2) Due to the characteristics of the tourism supply chain, the benefit distribution mechanism in the tourism supply chain alliance will be affected by the dual influence of tourism resource monopoly factors and time factors, so the benefit imbalance is inevitable in the process of benefit distribution. At this time, the stakeholders’ perception of the fairness of the benefit distribution process determines the stability of the supply chain alliance. The stakeholders’ perception of fairness is embodied by a sense of relative deprivation.
(3) In the case of imbalance of benefits, most of the groups with impaired benefits will actively demand the restoration of their due benefits, while some groups with strong benefits will weaken their tendency to profit out of consideration for the continuous cooperation of the alliance and their own reputation building. At the same time, external policy intervention will also coordinate the distribution of benefits within the alliance.

The dynamic variability of the all-for-one tourism supply chain alliance makes it put forward high requirements for the adaptability of benefit distribution. In view of the adaptive distribution flexibility of the ant colony labor division model, this paper applies the ant colony labor division model to the benefit distribution of alliance members, establishes the mapping relationship between the model and benefit distribution, combines the characteristics of member companies and fairness requirements, and expands the basic ant colony labor division model. On this basis, an extended ant colony labor division model for the benefit distribution problem of the all-for-one tourism supply chain is proposed.

## 4 Construction of an extended ant colony labor division model of benefit distribution in the all-for-one tourism supply chain

### 4.1 Fixed response threshold model of the ant colony labor division

The fixed response threshold model (FRTM) of the ant colony labor division was proposed by Bonabeau et al. (1996) on the basis of in-depth observation and the study of the adaptive dynamic task assignment behavior of ant colonies without leadership. The model is briefly described as follows: In the form of stimulus–response, each specific task in the environment has a corresponding stimulus value *s*. The urgency of the task is proportional to the magnitude of the stimulus value. The more urgent the task, the higher the stimulus value; otherwise, the lower it is. Similarly, according to whether its own ability is sufficient to solve the task, each ant has a fixed response threshold *θ* corresponding to the task, and the response threshold is a fixed value in the FRTM. The response threshold level of the individual ant reflects the actual difference in behavioral response; that is, the threshold determines the response state of the individual ant to a task. When the stimulus intensity of a task in the environment exceeds the response threshold of an ant, the probability of it engaging in the task is high; on the contrary, it has a low probability of performing the task. In addition, the stimulus intensity of the task will vary with the number and efficiency of performing ants. When the task is not executed by the ants, the stimulus value of the task will increase to stimulate more ants. When the ants performing a specific task withdraw from task execution, the intensity of the environmental stimulus corresponding to the task will increase until it reaches the level of ants with a higher response threshold, thereby inspiring these ants to start the task.

(1) The environmental stimulus value *s* varies with time

Environmental stimulus is a direct factor of the individual’s response to a task. When a task appears, the environment will produce a stimulus value to affect the individual’s state. The environmental stimulus value is proportional to the task amount. The larger the task amount, the greater the stimulus value, and *vice versa*.

The task will be completed gradually with the addition of the individual and the corresponding environmental stimulus will be gradually weakened. However, as long as the task is not completely completed, the environmental stimulus will keep increasing with the duration of the task, thus stimulating more individuals to participate in the task. The rules for increasing environmental stimuli are as follows:

$$s\left(t+1\right)=s\left(t\right)+\delta -\varphi \cdot n_{act},$$ (1)

where *t* is a discrete time variable, *s* (*t*) represents the environmental stimulus value at time *t*, *δ* represents the increase in unit time of the environmental stimulus, *φ* represents the amount of tasks completed by ants in unit time, and *n*_*act*_ represents the number of ants performing this task in unit time.

(2) Responses of non-performing ants to environmental stimuli

Once the task appears, the environment will give the ant a stimulus value and the ant *i* will determine the probability of participating in the task based on its own attribute characteristics.

$$P\left(S_{i}=0\to S_{i}=1\right)=\frac{s^{n}}{s^{n}+\theta_{i}^{n}},$$ (2)

where *s*_*i*_ represents the activity state of individual ant *i* and *s*_*i*_ = 1 represents the ant participating in performing a task. *s*_*i*_ = 0 indicates that the ant does not participate in the task. *P* (*S*_*i*_ = 0→*S*_*i*_ = 1) represents the probability that ant *i,* who has not performed the task, is affected by the environmental stimulus and changes from the non-executing state to the executing state, and *θ*_*i*_ is the response threshold of individual ant *i*. In the model proposed by Bonabeau et al. (1996), the index *n* is a constant controlling the curve shape of the threshold function, and in general, *n* = 2. It can be seen from Formula 2 that when *θ*_*i*_ is constant, the larger the *s* is, the larger the *P* is. This shows that the stronger the environmental stimulus is, the greater the probability of the ants responding to the task are, and when *s* is constant, the larger the *θ*_*i*_ is, the smaller the *P* is, indicating that the higher the threshold of the ant is, the smaller the probability of the individual responding to the task is.

(3) The probability that the performing ant quits the task

When ant *i* participates in task execution, after every period of time, it will decide whether to quit or not according to the following probability:

$$P\left(S_{i}=1\to S_{i}=0\right)=p$$ (3)

In the formula, *P* (*S*_*i*_ = 1→*S*_*i*_ = 0) represents the probability that the status of ant *i* performing the task changes from 1 to 0, that is, the probability of quitting the task. To simplify the model, *p* is generally a constant. In the FRTM, the probability of an ant participating in a task and the probability of quitting a task are independent of each other.

### 4.2 Similarity analysis between the supply chain alliance and ant colony labor division model

#### 4.2.1 Mapping relationship between the supply chain alliance and ant colony labor division model

**FIGURE 2:**
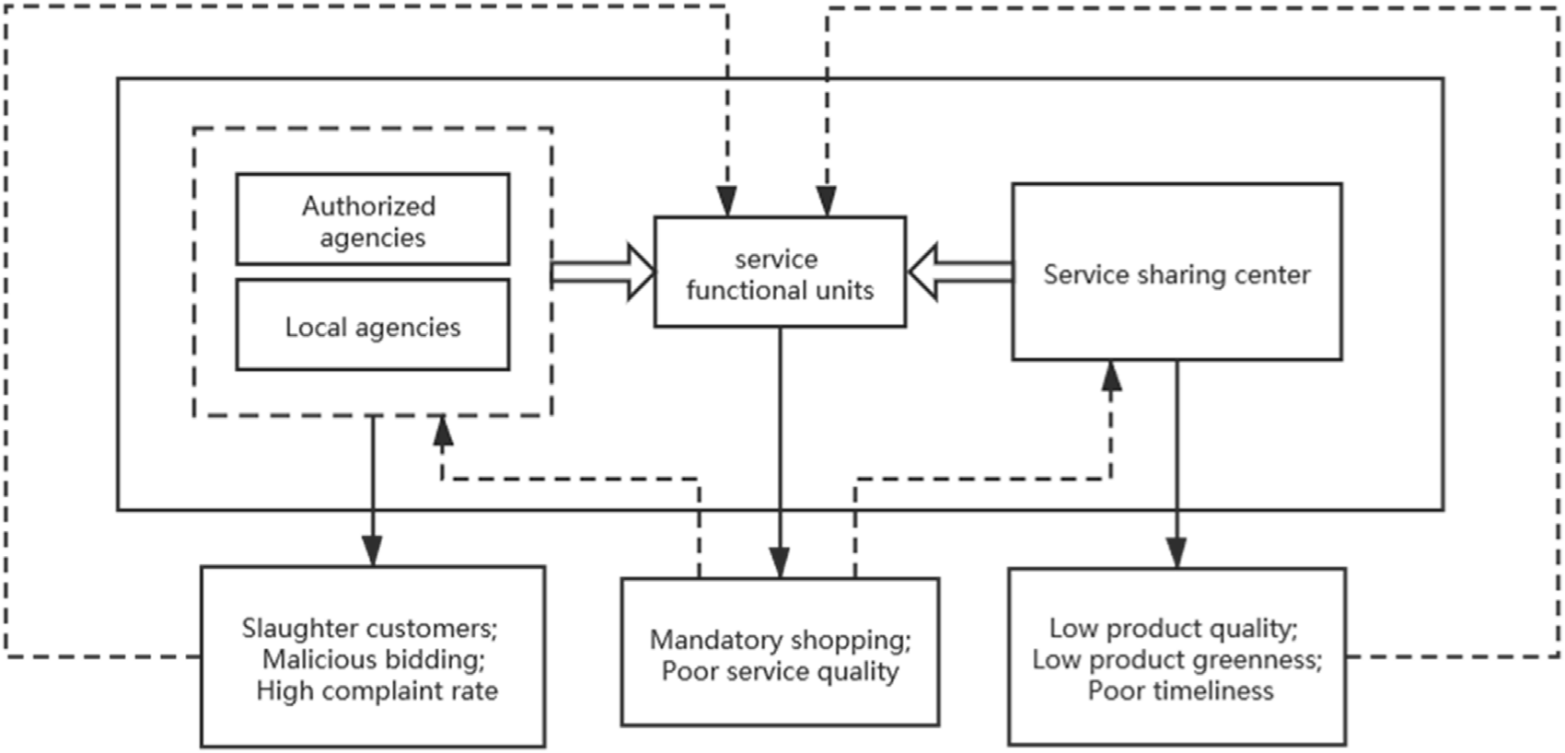
Membership of the tourism supply chain.

In the entire operation process of the all-for-one tourism scenic spot, member companies can be divided into tourism operators represented by travel agencies and tourism suppliers represented by service function units (hotels, restaurants, and entertainment places) and service sharing centers (SSCs) according to the different structures and scales. In order to maximize their efficiency, travel agencies usually outsource their business to local travel agencies at the destination. At this time, travel agencies can be divided into group travel agencies and ground travel agencies. Therefore, the membership in the supply chain alliance is shown in Figure 2. Among them, the close connection among travel agencies, service functional units, and SSCs makes any one of them only follow the principle of maximizing its own benefits without considering the coordination of the whole supply chain, which will cause certain losses to other subjects in the supply chain.

The mapping relationship between the benefit distribution of the all-for-one tourism supply chain and the ant colony labor division model can be summarized as follows:

(1) The member enterprises in the tourism destination supply chain are defined as ants, including service sharing centers, group travel agencies, and ground travel agencies
(2) The profitability of member enterprises is defined as the response threshold of ants to tasks
(3) The member enterprises' perception of benefit distributive justice is defined as environmental stimulus
(4) Both ants and members of the supply chain alliance decide whether to join the execution of the task or not according to the task and their actual ability
(5) Whether individuals and companies participate or not is spontaneous, and there is no mandatory contribution/membership/activity
(6) The characteristics of environmental stimulus changes over time in the ant colony labor division model are similar to the changes in perceptions of fairness by member companies in the all-for-one tourism supply chain alliance

The aforementioned analysis and explanation on the similarities between the two are aimed at demonstrating and clarifying the feasibility of research ideas in this article.

#### 4.2.2 Model extension ideas

The basic ant colony labor division FRTM is relatively simple, and the attributes of individual ant such as stimulus and threshold are simple too. If it is directly used in the modeling and simulation of tourism supply chain alliance benefit distribution, it will be difficult to reflect the complex relationship between the supply chain and the member enterprises. Therefore, this paper redesigned the basic attributes, response behavior, and benefit adjustment rules of ants. By comparing with the characteristics of tourism supply chain alliance, the basic ant colony labor division model was extended from the following aspects:

(1) In the basic ant colony labor division model, the threshold of ants remains fixed once determined, which obviously cannot fully fit the complex benefit distribution process. In this paper, the inhibition factor and the government subsidy factor are considered to coordinate the benefit distribution process of the supply chain alliance so that the response threshold of member enterprises will change in the changing environment, and then, it is more in line with the market benefit coordination mechanism.
(2) In the basic ant colony labor division model, the environmental stimulus value of the task is determined by the corresponding task quantity. However, in the tourism supply chain alliance, because the contribution size, perception of fairness, and requirements for the stability of the alliance of different member enterprises are different, it is necessary to consider that different member enterprises have different incentives to increase or decrease the benefit behavior.
(3) In the basic ant colony labor division model, there are only two states of ant joining and exiting, and the probability of ant quitting is usually a preset value, which does not meet the actual requirements. In the benefit distribution of the tourism supply chain alliance, it is necessary to consider the three situations of increasing, decreasing, and unchanged benefit of member enterprises, and the corresponding probability model should be constructed.

### 4.3 Benefits and its expressions

#### 4.3.1 Contribution

Different social roles and capabilities of enterprises lead to great differences in their contribution levels they make in the process of creating total social benefits. Their contribution can be measured by their investment in transportation, labor, facilities, equipment, technology, environmental protection, and other indicators in the process of creating the total benefits of the tourism supply chain. The contribution rate of member enterprise *i* can be expressed as

$$c_{i}=\frac{C_{i}}{\sum_{i=1}^{N}C_{i}},$$ (4)

where *C*_*i*_ represents the contribution of member *i* in the supply chain and *N* represents the number of members in the alliance.

#### 4.3.2 Expression of benefit

Member enterprise *i* generates the expected value *Q*_*i*_ for the distribution of total benefit according to its contribution in transportation, labor, facilities, equipment, technology, environmental protection, and other aspects, and *Q*_*i*_ = *c*_*i*_·*G* (*G* represents the total benefit and *G*_*i*_ represents the actual benefit of member enterprise *i*). When *G*_*i*_ = *Q*_*i*_, the expected benefit of member enterprise *i* matches the actual benefit, which is the most fair and reasonable distribution method. When *G*_*i*_ *< Q*_*i*_, the actual benefit of some member enterprises is lower than the expected benefit. Since the total benefit is fixed, that is, *G* = ∑*G*_*i*_ = ∑*Q*_*i*_, it can be known that the actual benefit of some member enterprises must be higher than the expected benefit, that is, *G*_*i*_ *> Q*_*i*_. As a result, the distribution of benefits is not balanced, which causes the dissatisfaction of member enterprises and then affects the service level and tourist experience of the all-for-one tourism scenic spot.

The gap between the actual benefit and the expected benefit of member enterprise *i* is defined as the degree of realization of the expected benefit *D*_*i*_; then,

$$D_{i}=\frac{G_{i}}{Q_{i}}.$$ (5)

The total degree of realization of the expected benefit is

$$TD_{i}=\sum_{i=1}^{N}D_{i}.$$ (6)

#### 4.3.3 Relative deprivation

Relative deprivation is a social psychological theory about group behavior, which was proposed by sociologist Stouffer (Smith et al., 2012; Flippen, 2013; Pettigrew, 2015) in 1949. This theory holds that some individuals will form a perception of their inferior status relative to the reference group after comparing themselves with the reference group and then produce negative emotions of “anger, resentment, and dissatisfaction.” These unbalanced psychological feelings are the main factors that trigger social unrest.

Drawing on the research of Xiao and Wang (2016) on the quantitative calculation formula of relative deprivation under the group benefit, the relative sense of deprivation generated by member enterprise *i* after comparing the realization degree of its expected benefits with the reference group is defined as

$$RD_{i}=\frac{AD\left(D_{i}\right)}{N},$$ (7)

where *AD* (*D*_*i*_) represents the sum of the parts in the reference group whose expected benefit degree value is higher than *D*_*i*_. *N* represents the number of members in the supply chain alliance.

### 4.4 Construction of the model of expanding the ant colony labor division

According to the applicability and defects of the ant colony labor division FRTM applied to the aforementioned benefit distribution of the tourism supply chain alliance, this paper proposes a new dynamic threshold model applied to the benefit distribution problem. The specific derivation process is as follows:

(1) Environmental stimulus

Environmental stimulus values reflect the response of ants to different tasks. The stronger the task stimulates, the more likely the ant is to engage in the task, and the weaker the task stimulates, the less likely the ant is to engage in the task. In the benefit distribution of the all-for-one tourism supply chain, the member enterprises have three behavioral choices, namely, increase in benefit, decrease in benefit, and unchanged benefit, which correspond to three behavioral stimuli, respectively. As the total benefit is fixed, the benefit of some member enterprises increases, which will inevitably lead to the benefit reduction of some other member enterprises. When the member enterprises' contribution is greater than their benefit and their sense of relative deprivation is high, they have a greater tendency to pursue benefit increase. At the same time, member enterprises sometimes choose to reduce their benefits based on their long-term development or establishing a good reputation. The change of member enterprises' selection tendency is related to the change of benefit and the factors of inhibition and subsidy.

The environmental incentives for member enterprises to increase their benefits are as follows:

$${s_{i}}^{+}=\frac{C_{i}}{C_{i}+G_{i}\left(t\right)},$$ (8)

where *C*_*i*_ is the contribution of member enterprise *i* in the supply chain and *G*_*i*_ is the actual benefit of member enterprise *i* at time *t*. It can be seen from Formula 8 that under the condition that the contribution level of a member enterprise remains unchanged, the higher its actual benefit, the smaller the incentive to increase the benefit, and the lower the actual benefit, the greater the incentive to increase the benefit. Under the circumstance that the actual benefit of a member enterprise remains unchanged, the higher its initial contribution level, the greater the incentive to choose the behavior of increasing benefit, and the lower its initial contribution level, the less the incentive to choose the behavior of increasing benefit.

In order to create a healthy tourism environment under the reasonable profit-making competition mechanism and the individual consideration that some subjects emphasize win–win cooperation over independent profit, some member enterprises will also have a tendency to reduce their benefits. The corresponding environmental incentives to reduce their benefits are

$${s_{i}}^{-}=\frac{G_{i}\left(t\right)}{G_{i}\left(t\right)+C_{i}}$$ (9)

It can be seen from Formula 9 that under the condition that the actual benefit of a member enterprise remains unchanged, the higher its contribution level, the smaller the incentive to reduce benefits; conversely, the greater the incentive to reduce benefits. Under the circumstance that the contribution level of a member enterprise remains unchanged, the higher the actual benefit, the greater the incentive to choose the behavior of benefit reduction; on the contrary, the less the incentive to choose the behavior of benefit reduction. The stimulus of increased and decreased benefits collectively reflects the negative correlation between profit and contribution of member firms.

(2) Stimulus *s* changes over time

In the basic ant colony labor division model, when the task is not fully completed, the environmental stimulus value will increase by a constant at every moment so as to stimulate more ants to participate in the task. With the continuous addition of ant individuals, the task is gradually decomposed and completed, and the environmental stimulus of the task will gradually weaken until it disappears. In the process of benefit distribution, in general, when the actual benefit is greater than the expected benefit, the willingness of member enterprises to continue to increase the benefit will be weakened, and the willingness to reduce the benefit will be relatively enhanced. However, when the actual benefit is lesser than the expected benefit, the willingness of member enterprises to continue to reduce the benefit will be weakened, and the willingness to increase the benefit will be relatively enhanced. The target task of each member enterprise is defined as the actual benefit equals to the expected benefit, that is, *D*_*i*_ = 1. When *D*_*i*_ > 1, the process of environmental stimulus of member enterprises over time is as follows:

$${s_{i}}^{+}\left(t+1\right)={s_{i}}^{+}\left(t\right)-\delta ,$$ (10)

$${s_{i}}^{-}\left(t+1\right)={s_{i}}^{-}\left(t\right)+\delta ,$$ (11)

When *D*_*i*_ *<* 1, the process of environmental stimulus of member enterprises over time is as follows:

$${s_{i}}^{+}\left(t+1\right)={s_{i}}^{+}\left(t\right)+\delta ,$$ (12)

$${s_{i}}^{-}\left(t+1\right)={s_{i}}^{-}\left(t\right)-\delta ,$$ (13)

where *δ* is the independent variable of environmental stimulus.

(3) Dynamic response threshold

The response threshold is an internal factor that determines whether member enterprises respond to stimulus *s* and make behavioral choices. The higher the response threshold of a member enterprise to increase its benefits, the lower its probability of taking benefit-increasing behaviors. Similarly, the higher the response threshold of a member enterprise’s benefit-reducing behavior, the lower its probability of taking benefit-reducing behaviors. The response threshold of member enterprises is determined by their profitability. In this paper, the functional relationship between the profitability of member enterprises and the response threshold is established. The response threshold of member enterprises to increase their benefits is

$$\theta^{+}=\lambda e^{-\frac{EGC_{i}*\kappa {\left(n\right)}^{\rho }}{\sum_{i=1}^{N}EGC_{i}}},$$ (14)

where *λ* is the threshold control coefficient to ensure the comparability between the threshold and the stimulus. *EGC*_*i*_ indicates the profitability of member enterprise *i* in the current all-for-one tourist attractions, which is determined by factors such as the size of the member enterprises, the social division of labor undertaken, and the degree of dependence on the season. *κ*(*n*) is an inhibitory factor, used to weaken the benefit realization ability of member enterprises that continues to increase benefits when *D*_*i*_ is greater than 1, and chooses benefit reduction behaviors for *n* consecutive times but *D*_*i*_ is still greater than 1. Taking into account the timeliness of benefit distribution, *n* is 3 here. *ρ* represents the number of times that *D*_*i*_ is still greater than 1 when it chooses the benefit reduction behavior for *n* consecutive times. *N* is the number of members in the supply chain alliance.

The response threshold of member enterprises to reduce benefits is

$$\theta^{-}=\frac{1}{\lambda }e^{\frac{EGC_{i}*\omega {\left(n\right)}^{\nu }}{\sum_{i=1}^{N}EGC_{i}}},$$ (15)

where *ω*(*n*) is an enhancement factor, which is used to enhance the benefit realization ability of member enterprises that continues to reduce benefits when *D*_*i*_ is less than 1 and choose the behavior of increasing benefits for *n* consecutive times but *D*_*i*_ is still less than 1. Similarly, *n* = 3, and *υ* refers to the number of times when *D*_*i*_ is still less than 1 when it chooses the behavior of increasing benefits for *n* consecutive times.

(4) Probability of behavioral choice

In the basic ant colony labor division model, ants perceive all environmental stimuli with equal probability. The sum of the selection probabilities of the ants that have not performed the task to participate in the task and the non-participation of the task is 1, and the sum of the selection probabilities of the ants that perform the task to exit the task and continue to perform the task is 1. In the model of this article, there are three different options: increase in benefit, decrease in benefit, and unchanged benefit. We define the probability of profit increase as *P* ^*+*^, the probability of profit decrease as *P* ^*−*^, and the probability of unchanged profit as *P* ^*^, and then, *P* ^*+*^+ *P* ^*−*^ + *P* ^*^ = 1.

The probability that member enterprise *i* responds to the increase in benefit is

$${P_{i}}^{+}=\frac{{\left(s_{i}^{+}\right)}^{n}}{{\left(s_{i}^{+}\right)}^{n}+{\left(\theta_{i}^{+}\right)}^{n}}=\frac{{\left[\frac{C_{i}}{C_{i}+G_{i}\left(t\right)}\right]}^{n}}{{\left[\frac{C_{i}}{C_{i}+G_{i}\left(t\right)}\right]}^{n}+\lambda^{n}e^{-\frac{EGC_{i}*\kappa {\left(n\right)}^{\rho }*n}{\sum_{i=1}^{N}EGC_{i}}}}.$$ (16)

The probability that member enterprise *i* responds to the decrease in benefit is

$$P^{-}=\frac{{\left(s_{i}^{-}\right)}^{n}}{{\left(s_{i}^{-}\right)}^{n}+{\left(\theta_{i}^{-}\right)}^{n}}=\frac{{\left[\frac{G_{i}\left(t\right)}{G_{i}\left(t\right)+C_{i}}\right]}^{n}}{{\left[\frac{G_{i}\left(t\right)}{G_{i}\left(t\right)+C_{i}}\right]}^{n}+\lambda^{-n}e^{\frac{EGC_{i}*\omega {\left(n\right)}^{\nu }*n}{\sum_{i=1}^{N}EGC_{i}}}},$$ (17)

The probability that member enterprise *i* responds to the unchanged benefit is

$$P_{i}^{*}=1-P_{i}^{+}-P_{i}^{-}.$$ (18)

(5) Change in benefit

$$\varepsilon^{+}=s_{i}^{+}\times EGC_{i}\times \left(H_{1}+H_{2}\cdot \kappa +H_{3}\cdot \omega \right)\times rand\left(\tau_{1}\right),$$ (19)

$$\varepsilon^{-}=s_{i}^{-}\times \frac{rand\left(\tau_{2}\right)}{EGC_{i}\times \left(H_{1}+H_{2}\cdot \kappa +H_{3}\cdot \omega \right)},$$ (20)

In Formulas 19, 20, *H*_1_, *H*_2_, and *H*_3_ are all decision variables. *H*_1_ = 1 and *H*_2_ and *H*_3_ = 0 when the member enterprise is not coordinated by the inhibitory factor *κ* and the enhancement factor *ω*. *H*_2_ = 1 and *H*_1_ and *H*_3_ = 0 when member enterprises are coordinated by the inhibitory factor *κ*. *H*_3_ = 1 and *H*_1_ and *H*_2_ = 0 when member enterprises are coordinated by the enhancement factor *ω*. *EGC*_i_*κ and *EGC*_*i*_ *ω reflect the benefit realization ability of member enterprise *i* under its self-inhibition and external intervention. *τ*_1_ is the average value of increased profits of member enterprises, and *rand* (*τ*_1_) stands for a random number between 0 and *τ*_1_. *τ*_2_ is the average value of reduction profit of member enterprises, and *rand* (*τ*_2_) stands for a random number between 0 and *τ*_2_ (Xiao and Wang, 2016). As shown in Formula 19, the stronger the incentive for a member enterprise to increase its benefits, the stronger the ability to realize its benefits and the greater the increase in its benefits. As shown in Formula 20, the stronger the incentive for a member enterprise to reduce its benefits, the weaker the ability to realize its benefits and the greater the reduction in its benefits.

Correspondingly, the benefits of member enterprises after increasing and decreasing benefits are

$$G_{i}\left(t+1\right)=G_{i}\left(t\right)+\varepsilon^{+},$$ (21)

$$G_{i}\left(t+1\right)=G_{i}\left(t\right)-\varepsilon^{-},$$ (22)

### 4.5 Algorithm implementation

Step 1 The initial discrete simulation time variable *T* = 0 and *T*_max_ as the maximum number of runs are set. The total benefit *G* is set, and the actual benefit *G*_*i*_ of member enterprise *i*, environmental stimulus independent variable *δ*, profitability *EGC*_*i*_, threshold control factor *λ*, inhibitory factor *κ* (*n*), and government subsidy factor *ω* (*n*) are initialized.

Step 2 The contribution rate of member enterprise *i* is calculated according to Formula 4. According to Formulas 5ormulas –Formulas 7, the degree of realization of expected benefits and relative deprivation is calculated.

Step 3 The environmental stimulus value and response threshold are calculated according to Formulas 8, 9, 14, 15.

Step 4 The individual behavior selection probability *P* ^*+*^, *P* ^*−*^, and *P* ^*^ are calculated according to Formulas 16ormulas –Formulas 18.

Step 5 The individual benefit *G*_*i*_ is updated according to Formulas 19ormulas –Formulas 22.

Step 6 The expected benefit realization degree *D*_*i*_, *TD*_*i*_, and the relative deprivation *RD*_*i*_ are updated according to Formulas 5ormulas –Formulas 7.

Step 7 The individual environmental stimulus value *s* (*t* + 1) is updated according to Formulas 10ormulas –Formulas 13. If *TD*_*i*_ =*N*, go to Step 8; otherwise, go to Step 3.

Step 8 Statistics and output the simulation results.

### 4.6 Simulation experiment and result analysis

#### 4.6.1 Problem background and parameter settings

City *R* is a national tourism demonstration zone city. In response to the call of the local government to deepen all-for-one tourism and accelerate the development of tourism, as well as the self-requirement of promoting the upgrading and transformation of the local tourism industry, City *R* has completed the comprehensive upgrading of the tourism supply chain, transforming the traditional independent node relationship of profitability into a win–win alliance relationship. Under the goal of sustainable development of tourism destinations, a new SSC node is added to the supply chain. The tourism supply chain alliance is generally composed of tourism operators and tourism suppliers. At present, a total of four enterprises have established alliance partnerships with one SSC, two travel suppliers represented by service function units, and one travel operator represented by travel agencies, which are, respectively, represented by Agent 1, Agent 2(a), Agent 2(b), and Agent 3.

This paper takes the benefit distribution of 10 tourism supply chain alliances as an example to verify the feasibility of the model. The total benefit and initial benefit of the 10 alliances are given in Table 1. Meanwhile, the expected benefit and relative sense of deprivation of the four enterprises in the initial state can be calculated. Other parameters are set as follows: The maximum number of runs *T*_max_ = 100 times, the environmental stimulus variable *δ* = 0.1, the contributions of the three groups are 68, 75, and 57, and the profitability *EGC*_*i*_ is 10, 7, and 3, respectively. The inhibitory factor *κ* = 0.4, and the enhancement factor *ω* = 1.1.

**TABLE 1:** 10 groups of benefit distribution of four companies in the initial state.

Number of benefit distribution		1	2	3	4	5	6	7	8	9	10
Total revenue (10,000 Yuan)		100	150	130	70	200	120	170	145	90	185
Initial revenue (10,000 Yuan)	Agent 1	53	77	55	39	88	61	83	72	51	92
	Agent 2(a)	26	39	41	17	36	33	46	39	23	49
	Agent 2(b)	14	18	22	11	58	15	24	18	11	28
	Agent 3	7	16	12	3	18	11	17	16	5	16
Expected revenue (*Q*_*i*_)	Agent 1	45	67.5	58.5	31.5	90	54	76.5	65.25	40.5	83.25
	Agent 2(a)	20	30	26	14	40	24	34	29	18	37
	Agent 2(b)	20	30	26	14	40	24	34	29	18	37
	Agent 3	15	22.5	19.5	10.5	30	18	25.5	21.75	13.5	27.75
Degree of expected realization (*D*_*i*_)	Agent 1	1.18	1.14	0.94	1.24	0.98	1.13	1.08	1.10	1.26	1.11
	Agent 2(a)	1.30	1.30	1.58	1.21	0.90	1.38	1.35	1.34	1.28	1.32
	Agent 2(b)	0.70	0.60	0.85	0.79	1.45	0.63	0.71	0.62	0.61	0.76
	Agent 3	0.47	0.71	0.62	0.29	0.60	0.61	0.67	0.74	0.37	0.58
Relative deprivation *RD* (*D*_*i*_)	Agent 1	0.03	0.04	0.16	0.00	0.12	0.06	0.07	0.06	0.00	0.05
	Agent 2(a)	0.00	0.00	0.00	0.01	0.16	0.00	0.00	0.00	0.00	0.00
	Agent 2(b)	0.27	0.34	0.04	0.22	0.00	0.31	0.26	0.33	0.33	0.23
	Agent 3	0.44	0.25	0.32	0.60	0.38	0.32	0.29	0.24	0.51	0.36
Total relative deprivation		0.74	0.63	0.52	0.82	0.66	0.70	0.61	0.64	0.84	0.65

#### 4.6.2 Analysis of simulation results

**FIGURE 3:**
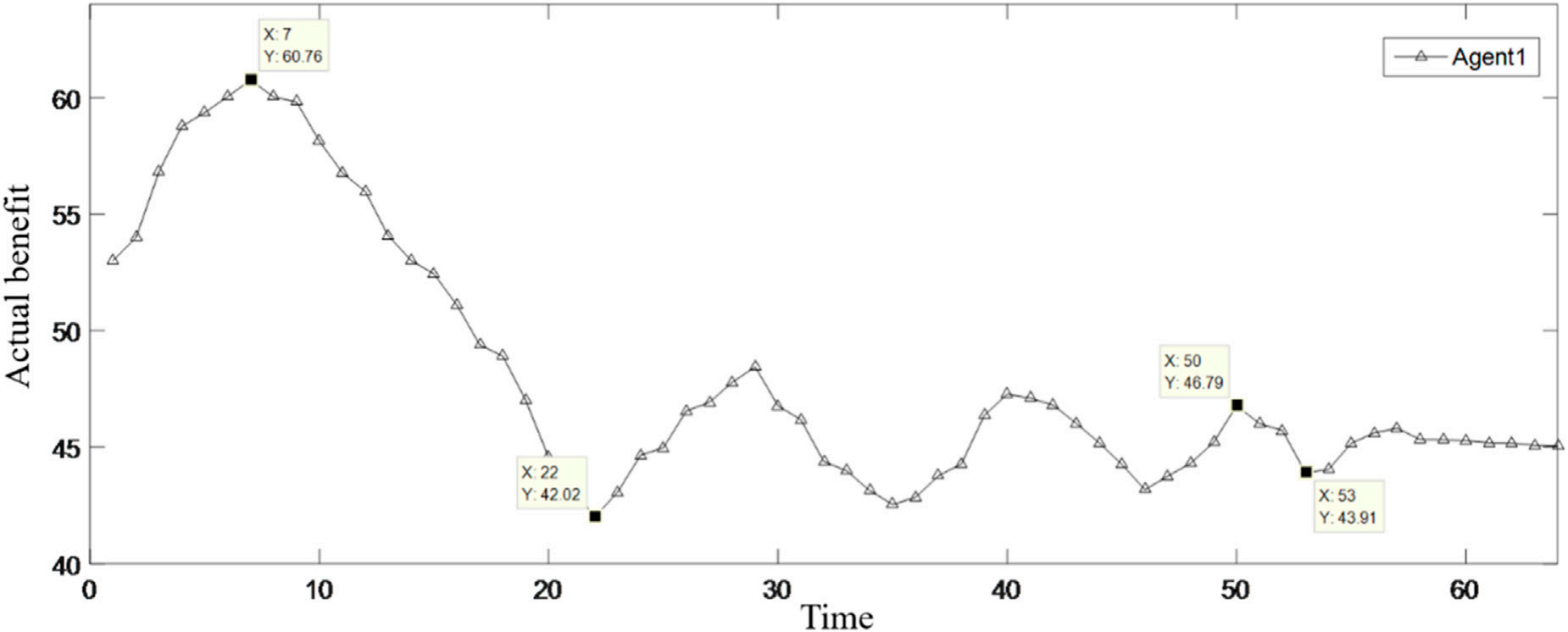
Changes in the benefits of Agent 1.

**FIGURE 4:**
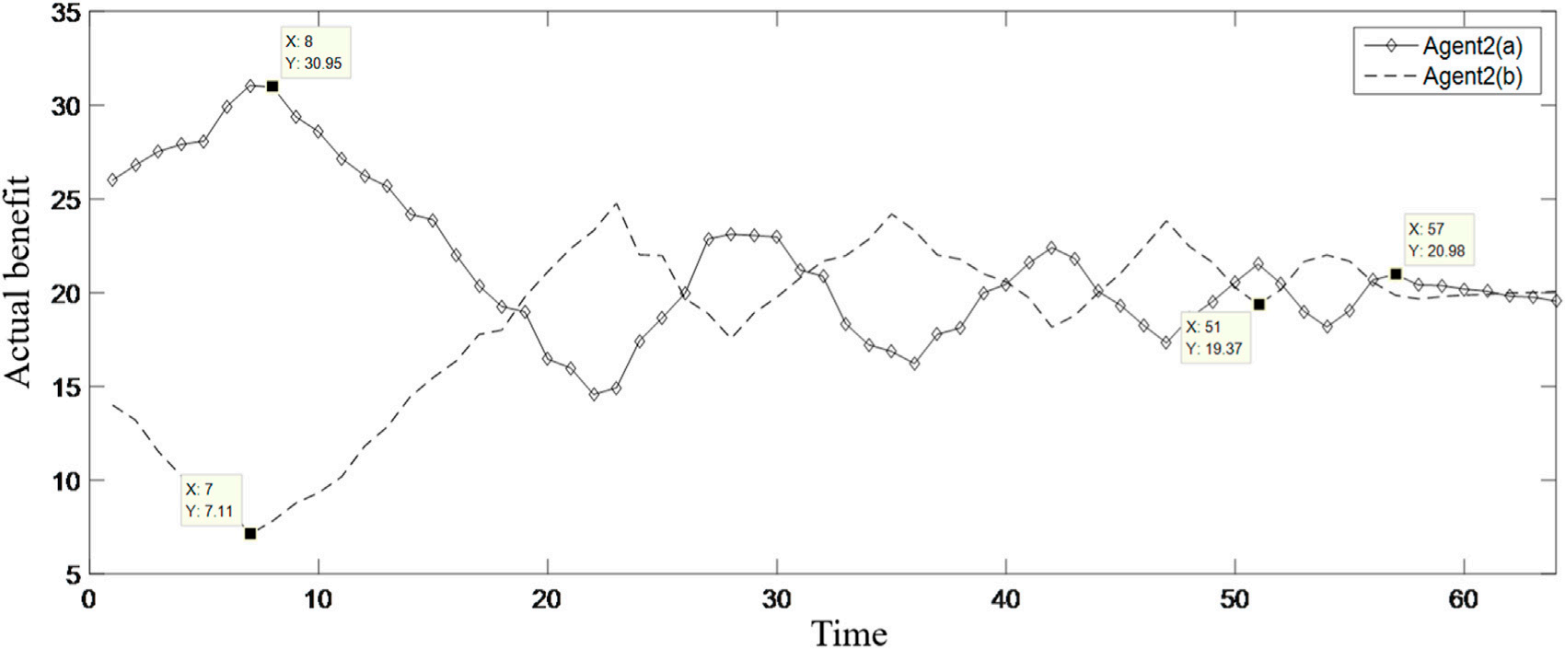
Changes in the benefits of Agent 2.

**FIGURE 5:**
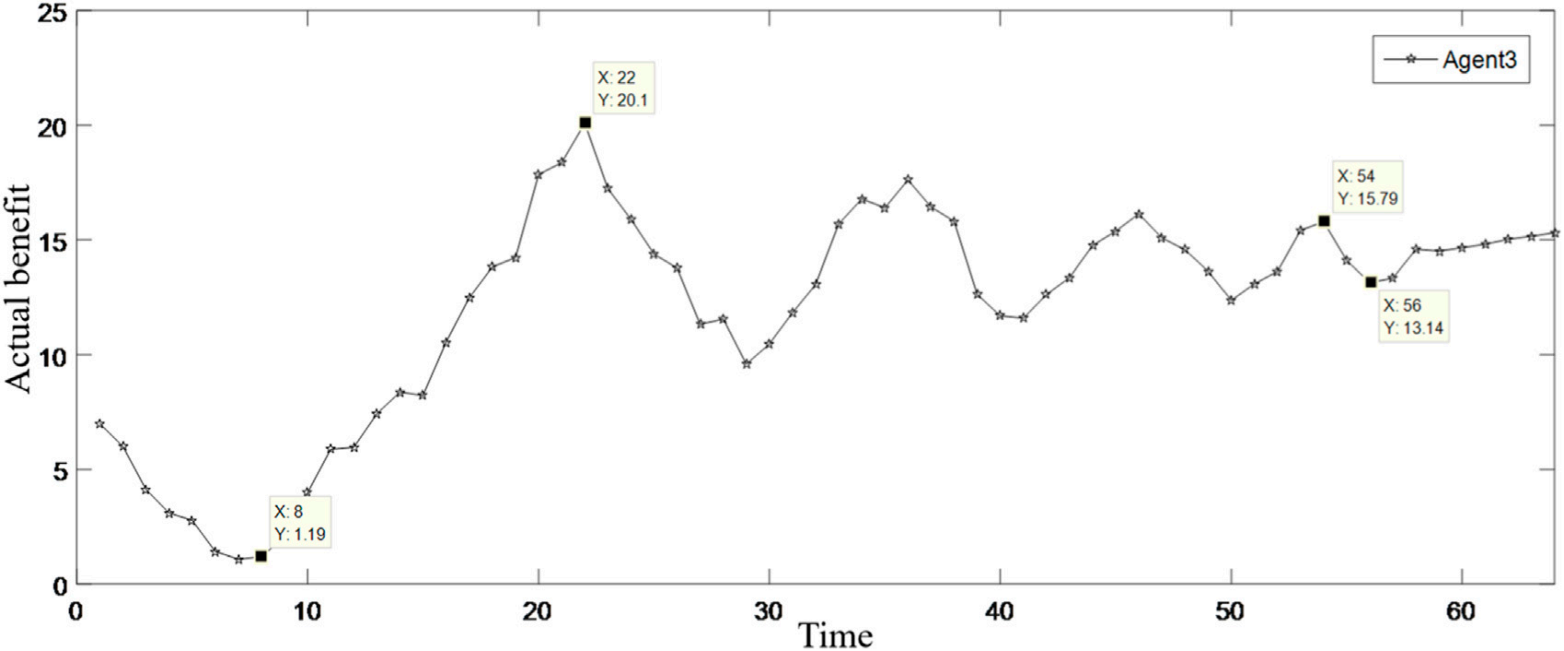
Changes in the benefits of Agent 3.

**FIGURE 6:**
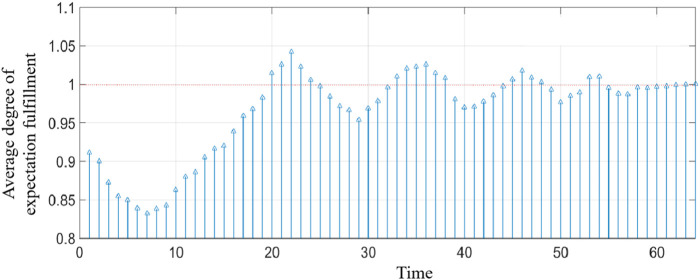
Average degree of the expected realization of member enterprises.

**FIGURE 7:**
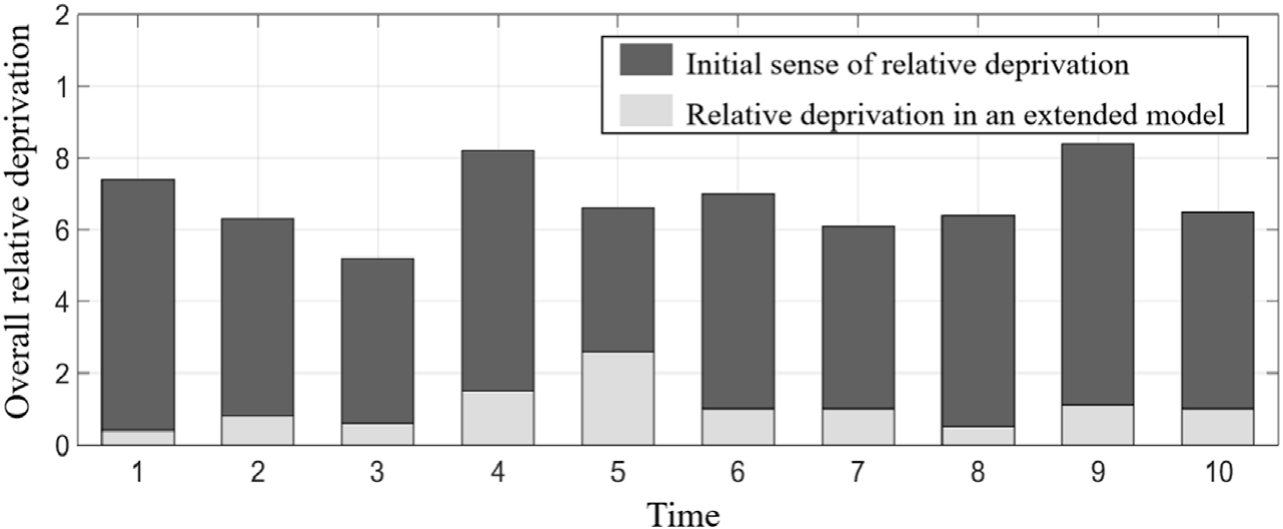
Total relative deprivation of member enterprises.

**FIGURE 8:**
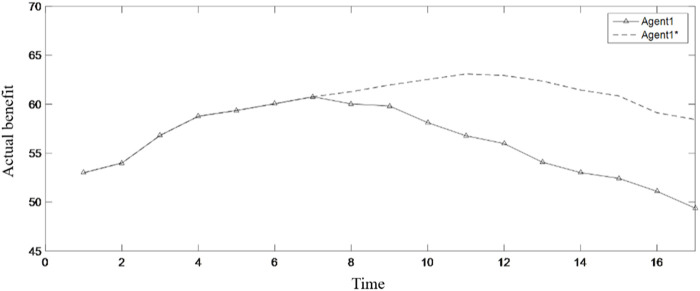
Impact of inhibitory factors on the actual benefits.

**FIGURE 9:**
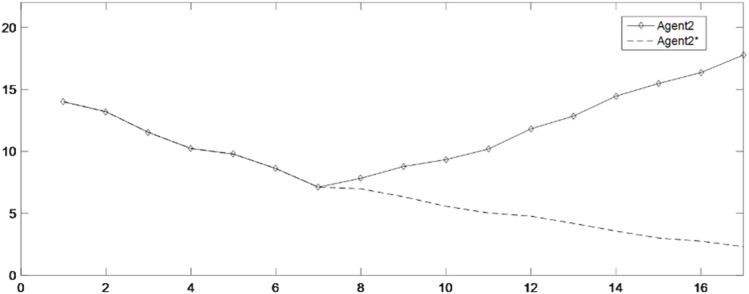
Impact of enhancement factors on the actual benefits.

MATLAB programming was carried out according to the established model, and the running results are shown in Table 2; Figures 3–9.

**TABLE 2:** 10 groups of benefit distribution based on the extended ant colony labor division.

Number of benefit distribution		1	2	3	4	5	6	7	8	9	10
Total revenue (10,000 Yuan)		100	150	130	70	200	120	170	145	90	185
Expected revenue (*Q*_*i*_)	Agent 1	45	67.5	58.5	31.5	90	54	76.5	65.25	40.5	83.25
	Agent 2(a)	20	30	26	14	40	24	34	29	18	37
	Agent 2(b)	20	30	26	14	40	24	34	29	18	37
	Agent 3	15	22.5	19.5	10.5	30	18	25.5	21.75	13.5	27.75
Revenue after distribution (10,000 Yuan)	Agent 1	45.06	68.23	57.71	29.48	91.04	52.23	75.12	64.13	39.83	84.71
	Agent 2(a)	19.55	29.84	27.04	15.52	39.69	23.71	35.83	30.22	17.02	38.59
	Agent 2(b)	20.08	31.06	26.3	13.71	41.27	26.44	32.28	28.48	19.49	36.24
	Agent 3	15.31	20.87	18.95	11.29	28	17.62	26.77	22.17	13.66	25.46
Degree of expected realization (*D*_*i*_)	Agent 1	1.00	1.01	0.99	0.94	1.01	0.97	0.98	0.98	0.98	1.02
	Agent 2(a)	0.98	0.99	1.04	1.11	0.99	0.99	1.05	1.04	0.95	1.04
	Agent 2(b)	1.00	1.04	1.01	0.98	1.03	1.10	0.95	0.98	1.08	0.98
	Agent 3	1.02	0.93	0.97	1.08	0.93	0.98	1.05	1.02	1.01	0.92
Relative deprivation *RD* (*D*_*i*_)	Agent 1	0.01	0.01	0.02	0.09	0.01	0.04	0.03	0.02	0.03	0.01
	Agent 2(a)	0.02	0.01	0.00	0.00	0.01	0.03	0.00	0.00	0.06	0.00
	Agent 2(b)	0.01	0.00	0.01	0.06	0.00	0.00	0.06	0.02	0.00	0.03
	Agent 3	0.00	0.06	0.03	0.01	0.24	0.03	0.00	0.01	0.02	0.07
Total relative deprivation		0.04	0.08	0.06	0.15	0.26	0.10	0.10	0.05	0.11	0.10

Table 2 shows the benefit distribution of the four enterprises in the 10 groups of experiments under the extended model. Compared with the benefit in the initial state in Table 1, the benefit gap of the alliance members with the ant colony labor division model has been significantly reduced. Taking the first set of experiments as an example, Figures 3–5 show the benefit changes of Agent 1, Agent 2(a), Agent 2(b), and Agent 3, respectively. It can be seen from Table 1 that the expected benefits of the four enterprises are 45, 20, 20, and 15, respectively. Combined with Figures 3–5, it can be seen that when the initial benefit of agents is relatively high, they still have the tendency to increase their benefit, and when the initial benefit of agents is relatively low, they still have the tendency to reduce their benefit due to the influence of their own capabilities. However, no matter whether the initial benefit of agents is high or low, in the process of program running, their benefit will be close to the expected benefit under the influence of their contributions. This phenomenon shows that under the effect of the benefit distribution model of expanded division of labor in ant colony, no matter what reasons the member enterprises gain or lose benefits at the early stage, they will finally realize the fair distribution of benefits under the demand of seeking the long-term stable development of the supply chain alliance.

Every change in the benefits of member enterprises is a game similar to bargaining. In the case of constant total benefit, member enterprises are inclined to increase their benefit as much as possible on the basis of maintaining the overall stable cooperation of the supply chain. With the increase in the number of games, the fluctuation of the benefits of the four enterprises gradually weakened. Taking Figure 3 as an example, the rising curve indicates that Agent 1 is willing to continuously increase its own benefit under the influence of environmental stimuli. However, as time increased, the inhibitory factors began to take effect, and Agent 1 slowly turns from increasing benefit to decreasing benefit. When the actual return falls below the expected return, Agent 1 turns to increase its own benefit. However, due to the great impact from the stimulation of reduced benefit in the previous moments, Agent 1 cannot achieve the balance between its expected benefit and actual benefit at one time, so it will adjust its return slowly and continuously according to environmental stimuli and external factors, and the cycle will not stop until its actual return is roughly equal to the expected return.

The moments X = 7, 22, 50, and 53 in the figure are taken as examples. When X = 7, Agent 1 reaches the maximum value of benefit under the action of its own will. In order to maintain stable cooperation in the supply chain, the inhibiting factor starts to work. It can be seen from the figure that after the inhibitory factor takes effect, the first time, the benefit of Agent 1 drops to the lowest point 42.02 and the last time, the benefit drops to the lowest point 43.91. It shows that as the number of games increases, the stimulus for Agent 1 to reduce its benefit becomes smaller each time, and the manifestation is that the reduction is getting smaller. The return of Agent 1 at the first time increase to the maximum point is 60.76, and the return at the last time increase to the maximum point is 46.79, indicating that with the increase in the number of games, the incentive for Agent 1 to increase the return each time will become smaller, which is manifested in the smaller increase range. In the cyclical game of increasing and decreasing benefit, Agent 1 gradually achieves the balance between expected benefit and actual benefit, which shows that as the number of iterations increases, the model runs better.

Figures 6 and 7, respectively, show the average expected realization degree and the overall relative deprivation of member enterprises. It can be seen from Figure 6 that, as the number of games increases, the average expected realization degree of member enterprises gradually approaches 1, that is, the distribution of benefits in the alliance gradually takes the contribution of member enterprises as the distribution standard. At the same time, it can be seen from Figure 7 that the relative deprivation of member enterprises in the initial state is relatively high and fluctuates between 0.5 and 0.85 in all 10 groups of experiments, indicating that the fairness of benefit distribution in the tourism supply chain alliance is low. However, in the benefit distribution based on the extended ant colony labor division model, the overall relative deprivation is greatly reduced, indicating that the tourism supply chain alliance gradually realizes the equitable distribution of benefits under the effect of the expanded model. The aforementioned analysis reflects the feasibility of the model and the superiority of solving the problem of benefit distribution.

In the game process of benefit increase and decrease, the member enterprises are jointly affected by the internal effects of the environmental stimulus and the external effects of inhibiting factors and enhancement factors, which is manifested in the different incentives to increase or decrease benefits with the change of their own actual benefits and expected degree of realization. When the benefit increases, the member enterprises have the willingness to reduce the benefit; and when the benefit decreases, the member enterprises have the willingness to increase the benefit. On the other hand, in order to maintain the long-term stable cooperation of the tourism supply chain alliance, apart from relying on the self-adjustment of the alliance members, a certain form of benefit distribution mechanism can also be established within the alliance.

Considering the supervision role of the government and public departments in the all-for-one tourism supply chain, the model in this paper introduces inhibiting factors and enhancing factors to conduct some external intervention in the benefit distribution process. Taking the changes in the benefits of Agent 1 and Agent 3 as an example, Figure 8 shows the inhibitory effect of inhibitory factors on the powerful benefit distribution group Agent 1. Among them, the dotted line is the benefit variation process of Agent 1 without the intervention of inhibitors, while the solid line is the benefit variation process of Agent 1 with the intervention of inhibitors. It can be seen from Figure 8 that the inhibitory factor takes effect at the seventh moment. Under the intervention of the inhibitory factor, Agent 1 can quickly reduce its benefit to near the expected return. Then, as shown in Figure 3, Agent 1 starts to fluctuate up and down based on the expected return, while without the intervention of inhibitory factors, the powerful group attribute of Agent 1 would lead to its continuous increase in returns. Although the benefit begins to decrease at about the eleventh moment, the reduction speed is very slow and the reduction range is very small, which has little impact on the benefit distribution of the whole supply chain.

Figure 9 shows the enhancement effect of enhancement factors on the benefit-impaired group Agent 2(b). Among them, the dotted line is the benefit variation process of Agent 2(b) without the intervention of enhancement, while the solid line is the benefit variation process of Agent 2(b) with the intervention of enhancement. It can be seen from Figure 9 that under the intervention of enhancement factor, Agent 2(b) rapidly increases the benefit to near the expected return, and then, as shown in Figure 4, Agent 2(b) also starts to fluctuate up and down based on the expected return, while without the intervention of enhancement factors, the benefits of Agent 2(b) are exploited by other powerful groups, resulting in a continuous decrease in its benefits. Although Agent 2(b) will also have a tendency to increase benefit under the effect of the environmental stimulus, the same is that the growth rate is relatively weak. In the long run, the distribution of benefits in the supply chain will be in an unbalanced state.

The aforementioned analysis shows that the inhibitory factors and enhancement factors set by the model in this paper have a good effect on the pursuit of fairness and efficiency in the benefit distribution process.

## 5 Conclusion

In the all-for-one tourism supply chain, the most fair and ideal state is to distribute benefits according to the respective contributions of member enterprises. However, in the actual situation, the influence of the off-peak season, the degree of cooperation between the upstream and downstream of the supply chain, the social status of member enterprises, and the widespread speculation in the tourism supply chain are all likely to affect the final actual benefit. If a fair and reasonable benefit distribution mechanism is not established, it is likely to form a situation in the supply chain where the strong becomes stronger and the weak becomes weaker, which will cause an imbalance of benefit distribution in the whole supply chain and seriously affect the stability of the supply chain. In this paper, a benefit distribution model of the extended ant colony division of labor for all-for-one tourism supply chain alliance is established by introducing inhibitory factors and enhancement factors and taking the expected realization degree of member enterprises as the criterion of fairness. The results show the following:

(1) In several experiments with different initial benefits, the model in this paper can realize the fair distribution of benefits by setting the fairness criteria and double factors.
(2) Based on their own capabilities, there are groups with strong interests, intermediate groups, and disadvantaged groups in the alliance. In the absence of the benefit distribution mechanism, the strong groups always tend to increase their profits, while the weak groups can only choose to continue to reduce their profits in the exploitation. The unfair distribution of benefits can easily damage the stability of the alliance.
(3) Considering the long-term stable development of the alliance, some powerful groups will have the intention to reduce their own benefits, but the effect is not significant in the initial stage of benefit distribution of the whole alliance, but will become the main factor to maintain the stability in the later stages.
(4) The extended model considers the introduction of inhibitory factors and enhancement factors to act on the benefit distribution process, and the setting of this dual factor can quantitatively explain the intervention effect of regulatory authorities in reality. With a certain degree of intervention by the regulatory authorities, the fair and just distribution of benefits in the supply chain alliance can be realized quickly and efficiently.
(5) In order to facilitate the study, the model in this paper is based on the determination of demand and information symmetry in all aspects of the global tourism supply chain. Further research is needed on the specific effects of enhancing factors and inhibiting factors.

Based on the aforementioned analysis, this paper believes that the supply chain alliance can adopt the extended ant colony labor division model to distribute the overall benefits.

## Data Availability

The original contributions presented in the study are included in the article/Supplementary Material. Further inquiries can be directed to the corresponding author.
